# Attentional biases to signals of negative information: Reliable measurement across three anxiety domains

**DOI:** 10.3758/s13428-024-02403-6

**Published:** 2024-03-25

**Authors:** Julian Basanovic

**Affiliations:** 1 Department of Psychology, University of Exeter, Washington Singer Laboratories, Exeter, EX4 4QG UK; 2 School of Psychological Science, The University of Western Australia, Perth, Australia

**Keywords:** Attention, Attentional bias, Emotion, Anxiety, Fear

## Abstract

Cognitive models propose that individuals with elevated vulnerability to experiencing negative emotion are characterised by biased attentional responding to negative information. Typically, methods of examining these biases have measured attention to pictures of emotional scenes, emotional faces, or rewarding or feared objects. Though these approaches have repeatedly yielded evidence of anxiety-linked biases, their measurement reliability is suggested to be poor. Recent research has shown that attentional responding to cues *signalling* negative information can be measured with greater reliability. However, whether such biases are associated with emotion vulnerability remains to be demonstrated. The present study conducted three experiments that recruited participants who varied in trait and state anxiety (*N* = 134), social anxiety (*N* = 122), or spider fear (*N* = 131) to complete an assessment of selective attention to cues signalling emotionally congruent negative information. Analyses demonstrated that anxiety and fear were associated with biased attentional responding to cues signalling negative information, and that such biases could be measured with acceptable reliability (*r*_split-half_ = .69–.81). Implications for research on the relation between emotion and attention are discussed.

For decades, cognitive theories have proposed that individuals who display elevated vulnerability to experiencing negative emotional states, such as anxiety, fear, or low mood, are characterised by biased attentional preferences for emotionally negative information. These theories have been supported by research demonstrating that heightened anxiety is associated with greater attentional orienting towards negative information and greater subsequent attentional avoidance of negative information (Bar-Haim et al., 2007; Beck & Clark, 1997; Mathews & Mackintosh, 1998; Mogg & Bradley, 1998; Williams et al., 1988), that heightened depression is associated with reduced attention towards positive emotional information (Joormann & Gotlib, 2007; LeMoult & Gotlib, 2019; Winer & Salem, 2016), and that other emotional-linked domains such as body image (Dondzilo et al., 2021; Dondzilo & Basanovic, 2023) and chronic pain (Todd et al., 2015, 2018) are associated with biases favouring emotion-congruent negative information, such as “thin-ideal” bodies and pained facial expressions, respectively.

To investigate attention to emotional information, researchers have developed tasks that measure selective attentional allocation to emotional stimuli. The most common of these tasks is the “attentional-probe” or “dot-probe” paradigm (MacLeod et al., 1986, 2002). The paradigm repeatedly presents participants with pairs of visual stimuli on a screen. One stimulus in the pair depicts emotional information and the other depicts benign information. Following a brief duration, the stimuli are removed, and one stimulus is replaced by a visual target that participants must discriminate with a response. Under the assumption that participants will respond more quickly to targets presented in the location of the stimulus they had attended to, researchers compare response latencies for targets in each stimulus location to infer biases in the allocation of attention between each stimulus type.

In the case of anxiety, research has examined attentional responding to emotionally discrepant word pairs (e.g., “cancer”, “table”), negative and non-negative emotional scenes (Basanovic & MacLeod, 2017; Koster et al., 2007; Mogg et al., 2004; Rudaizky et al., 2014; Yiend & Mathews, 2001), angry and neutral facial expressions (Bradley et al., 1999; Mazidi et al., 2021; Pishyar et al., 2004), and feared and non-feared animals (Basanovic et al., 2017; Merckelbach et al., 1993; Rinck & Becker, 2006). It is notable, however, that anxiety-linked differences in attention to negative information are not consistent across anxiety domains. For example, meta-analytic evidence examining individuals who differ in trait anxiety have tended to demonstrate elevated trait anxiety to be associated with greater attention towards negative stimuli (Bar-Haim et al., 2007; Liu et al., 2019). Further, some studies have also indicated that variation in state anxiety can moderate the association between attention to negative stimuli and trait anxiety, such that heightened state anxiety results in greater attention to negative information in high trait-anxious individuals, as compared to low trait-anxious individuals (MacLeod & Mathews, 1988; Mogg et al., 1994). With respect to social anxiety, probe-based tasks have generally shown heightened social anxiety to be associated with heightened attention towards angry faces, as compared to benign faces (Bantin et al., 2016). While fewer in number, probe-based studies examining spider fear have revealed mixed evidence concerning attentional vigilance for spider-relevant stimuli (Lipp & Derakshan, 2005; Merckelbach et al., 1993; Mogg & Bradley, 2006), while continuous measures of eye gaze across durations of several seconds have indicated heightened spider fear to be associated with initial attentional orientation towards spider stimuli followed by sustained attentional avoidance (Hermans et al., 1999; Pflugshaupt et al., 2005; Rinck & Becker, 2006).

Though the attentional-probe paradigm has repeatedly revealed evidence of anxiety-linked biases in attentional responding to negative information, recent investigations have found the method to have poor reliability in its measurement. Reported split-half reliability estimates of the difference in mean response latencies for probes presented in each stimulus location, a measure commonly used to index biases in attentional responding, have typically ranged from *r* = −.26 to *r* = .35 (Basanovic et al., 2021; Chapman et al., 2017; Clarke et al., 2020; Schmukle, 2005; Van Bockstaele et al., 2019; Waechter & Stolz, 2015), well below the criterion of .70 that is commonly taken to indicate a sufficient level of measurement reliability for cognitive assessments in research (Ponterotto & Ruckdeschel, 2007). Importantly, poor measurement reliability has long been known to limit the capacity for researchers to accurately investigate hypotheses. Poor reliability reduces observable effect sizes, constrains the maximum observable association between variables, and reduces statistical power to detect true effects (Parsons et al., 2019; Schmidt & Hunter, 1999), impairs comparisons of effect sizes of different measures (Cooper et al., 2017), and impairs replication efforts (Shaw et al., 2020).

The importance for resolving poor measurement of attentional biases has been highlighted by mixed evidence concerning the presence of attentional biases in anxiety disorders. While there is evidence that greater anxiety vulnerability is associated with attentional biases for negative information (Bar-Haim et al., 2007), and that the manipulation of attentional biases can reduce symptoms of anxiety disorders (Hallion & Ruscio, 2011), recent meta-analytic evidence has indicated that individuals with clinical diagnoses of anxiety disorders do not exhibit an attentional preference for negative information (Kruijt et al., 2019). While one explanation is that clinical anxiety is not characterised by bias for negative information (and indeed this conclusion is not incompatible with research that has assessed differences in biased attention between anxious and non-anxious groups), another plausible explanation is that poor measurement reliability is resulting in “true” biases are not being detected in research. Critically, this ambiguity currently impairs the progress of research in understanding the role and potential utility of attentional biases in clinical disorders.

In contrast to research measuring attentional responding to negative information, some researchers have examined anxiety-linked differences in attentional responding to stimuli that *signal* forthcoming negative information. Though these signals do not contain emotional information themselves, attentional responding to such signals can reveal differences in attentional preferences for emotional information, because attention to such cues would be highly relevant for an attentional system geared towards responding to negative information in a particular way. For example, an anxious person may find their attention captured by a sound heard outside the window at night, a socially anxious person to an audience member’s movements as they present a talk, or a spider-fearful person to the presence of a spider’s web.

Evidence exists to suggest this is the case. Using a conditioning paradigm, Koster et al. (2005) observed that participants disproportionately allocated attention towards visual cues that signalled an imminent uncomfortable burst of noise as compared to cues that did not signal the noise. However, the investigators did not investigate whether attention to the cues was associated with emotional vulnerability. In other work, investigators have examined anxiety-linked differences in attention to cues signalling a financial loss or uncomfortable noise burst (Georgiades et al., 2021; Notebaert et al., 2017, 2020). Though these studies also incorporated experimental manipulations on the controllability of the negative outcome, they did observe that, in general, anxiety vulnerability was associated with heightened attention towards cues signalling the negative outcome. Unfortunately, however, these researchers did not test the reliability of the measurements obtained from these methods.

Studies have also demonstrated that individuals show heightened selective attention to cues signalling the presence of pictures of negative information, such as an angry face, and that such biases can be assessed with a higher level of measurement reliability than has been reported for the traditional attentional-probe task paradigm. Gladwin et al. (2019, 2020) presented participants with image pairs displaying a an angry face and a neutral face. Each image pair was preceded by coloured cues that indicated the imminent location of each face. On some trials, the coloured cues were followed by attentional probes in each location, instead of the face images. The investigators found that participants were quicker to discriminate probes presented in the location of cues that signalled the location of angry faces, as compared to neutral faces. This indicated that participants exhibited an attentional preference for cues that signalled negative information. The authors also demonstrated that an index of the difference in mean response latencies for probes presented in each stimulus location, providing a measure of the degree to which attention was biased, held greater levels of split-half internal reliability (*r* = .56 to .69). While this particular paradigm has not shown a link with anxiety vulnerability, it has revealed relationships between biased attention to cues signalling alcoholic beverages and individual differences in alcohol consumption behaviour (Gladwin, 2019; Gladwin et al., 2020). Other studies have used the cue-signal approach while manipulating the probability of the target location relative to cue locations to experimentally induce an attentional bias towards or away from cues signalling negative information (Gladwin et al., 2021). These revealed that anxiety vulnerability was associated with an attentional bias congruent with the inducement in blocks that sought to bias attention away from negative information, but not in blocks that sought to bias attention towards negative information. Together, these studies suggest that the cue-signal paradigm may be capable of revealing relationships between individual variation in attention to negative signals and emotional vulnerability if one exists.

The prospect that anxiety may be associated with biases in attentional responding to signals of negative information, and that such biases may be measured with an acceptable degree of reliability, provides the impetus for the present study. Determining whether anxiety vulnerability is associated with biases in attention to signals of negative information could inform cognitive models of attention and anxiety that have been predominantly based on research examining how anxiety biases attention in response to the presentation of negative information (Bar-Haim et al., 2007; Cisler & Koster, 2011). For example, if rigorous investigations reveal that anxiety is associated with biased attention to neutral signals of emotional information, this could indicate new mechanisms through which emotion interacts with attention, such as through processes that operate to proactively respond in anticipation of negative information, as compared to processes that operate to reactively respond in the presence of negative information. This could also benefit other emotion domains that consider attentional processing of emotional information, such as depression, body image, and chronic pain. Furthermore, determining whether such biases can be measured with an acceptable degree of measurement reliability will reveal whether measurement of this bias is appropriate for work striving to understanding the relation between attention and emotion.

For these reasons, the aim of the present study was to determine whether biases in attentional responding to cues signalling the imminent location of negative information are related to anxiety vulnerability, and whether such biases can be measured with acceptable reliability. Three experiments recruited participants who varied in either trait and state anxiety, social anxiety, or specific fear. Participants completed an attention assessment task that presented a pair of visual cues indicating the imminent location of negative information and non-negative information congruent with the individual difference of interest. On most trials, cues were accurately replaced by pictures depicting negative information (e.g., a negative scene, a negative facial expression, or a spider) and non-negative information. On remaining trials, one cue was replaced with a visual target that participants were required to discriminate. Biased attentional responding to cues signalling the imminent location of negative stimuli was inferred by comparing response latencies for probes replacing each cue. The relationship between anxiety vulnerability and biased selective attention to cues signalling negative pictures was analysed using a mixed-effects model approach.

The nature of any potential anxiety-linked biases and the reliability of their measurement was not hypothesised. However, it was predicted that any anxiety-linked difference in attentional responding to cues signalling negative information would be revealed through a moderating influence of anxiety vulnerability upon the difference in response latencies for probes presented in the location of cues signalling negative as compared to non-negative pictures.

## General method

Approval to conduct this research was obtained from the Human Research Ethics Committee of the University of Western Australia. Three experiments were conducted simultaneously, and each experiment had common methodological features in participant recruitment, task design, and procedure, though they differed with respect to the individual differences of interest and emotional information used. The features common across studies will be described first, followed by features unique to the design of each experiment and the results of each experiment.

## Method and data analysis common across experiments

### Participants

Participants were recruited from the psychology undergraduate participant pool of the University of Western Australia, who participated in the study in exchange for course credit. Participants were recruited over the course of four university teaching semesters at the university (April 2021–October 2022), and recruitment was ceased at a pre-determined date. Details of participants in each experiment are provided in the experiment-specific method sections.

### Attention assessment task

The attention assessment task was designed to measure the degree to which participants demonstrated biased attentional responding to visual cues indicating the imminent location of pictures depicting negative information, as compared to non-negative information. The task’s core feature of signalling information with neutral cue stimuli and measuring attention to these cues built on work described by Gladwin et al. (2019, 2020).

**Fig. 1:**
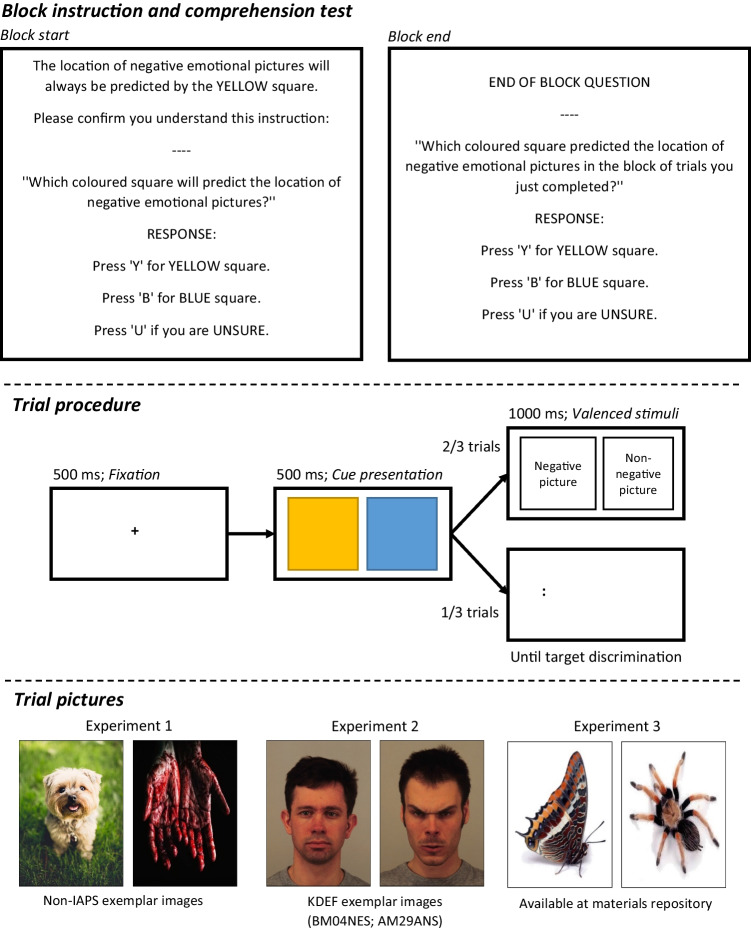
Illustration of block instruction trial procedure for the attention assessment task used in each experiment. Figure not to scale

The task presented participants with repeated trials during which coloured visual cues predicted the location of subsequently presented negative and non-negative pictures (“picture trials”). These trials served to demonstrate the association between cue colour and picture valence. Interspersed amongst these trials were trials that presented the same cues, but that probed attentional responding to the cues by instead following one of the cues with a visual target that participants were required to discriminate (“probe trials”). These trials were used to assessed relative attention allocation between the cues that predicted negative and non-negative information. An illustration of these trial types is present in Fig. 1.

Trials in the task were presented across a series of blocks. Each block commenced by informing the participant of the colour of a visual cue that would predict the location of negative and non-negative picture. Participants then completed a comprehension test for this information. At the end of each block, participants were asked to recall the colour of the negative picture cue. Each block comprised picture trials and probe trials in randomised order across each task block. Details of each trial type will now be described.

#### Picture trials

Two-thirds of the trials in each block were “picture trials”. These trials commenced with a fixation cross presented in the centre of the screen for 500 ms. Next, the cross was cleared, and two visual cues were presented on screen. The visual cues were a blue and yellow square, and the cue colour for each picture type was fixed across the task and counterbalanced across participants. One cue was presented to the left of screen centre, and one to the right of centre. The location of each cue was randomised, though each cue was presented in each location with equal frequency across trial blocks. Each cue was 85 mm in height and width, and the distance between the centres of the cues was 135 mm. The cues were presented for 500 ms, before being replaced by two pictures depicting negative information and non-negative information. The cues always accurately predicted the subsequent location of the pictures. The pictures were presented for 1000 ms, after which the screen was cleared. The next trial commenced after a 500 ms delay.

#### Probe trials

One-third of the trials in each block were “probe trials”. These trials commenced with a fixation cross presented in the centre of the screen for 500 ms. Next, the cross was cleared, and the same two cues were presented on screen. Following their presentation, a visual target was presented in the location of the negative picture cue or non-negative picture cue, at random but with equal frequency across trials. The target was two dots, distanced 5 mm apart, and aligned either horizontally ( . . ) or vertically ( : ). A foil presented a diagonal dot pair and was presented in the alternate location to reduce attentional capture by onset of a single stimulus. Participants were instructed to identify the target as quickly and accurately as possible, by pressing the “H” or “V” key on the keyboard. The target and foil remained on screen until participants responded. Upon a correct response, the screen was cleared. Upon an incorrect response, the word “INCORRECT” appeared on screen for 3 seconds. The next trial commenced after a 500 ms delay. The trial recorded the latency and accuracy of the target discrimination response. The latency at which participants discriminated targets in each cue location was used to measure attention to cues signalling negative pictures.

### Procedure

The procedure was delivered online via the participants’ personal device at a time and location of their choosing. Upon starting the study procedure, participants were provided with an information and consent form. Once consent was obtained, participants calibrated their monitor to a known spatial distance (the width of a credit card) which allowed the presentation software to maintain the spatial parameters of stimuli across different monitor sizes and resolutions. Participants next completed demographic information and experiment-specific questionnaires (described below). To adhere to informed consent requirements, participants then viewed eight pictures randomly drawn from the picture set used in the attention task before confirming their desire to continue. Participants next received instruction on the attention task and completed a practice block of 24 trials presenting pictures of abstract art in place of the picture set. Participants then completed the experiment critical trials of the attention task. Lastly, participants completed a questionnaire that asked them to confirm the integrity of their data for analysis. Responses on the integrity questionnaire did not influence reimbursement for participation. Upon completion of the procedure, participants were provided with reimbursement and debriefing information.

### Data analysis

Participant data was considered invalid and excluded from data analysis if it demonstrated an excessively long procedure duration (> 120 minutes), if the participant commenced the procedure more than once, or if the participant indicated that their data should not be used for analysis. Remaining participants were excluded from inferential analysis if the accuracy of their probe discrimination responses was below 90%, or if they failed a comprehension check for the colour of the cue stimulus that signalled negative pictures.

For valid participants, the latencies of incorrect responses and latencies less than 200 ms or greater than 2000 ms were eliminated. Next, for each participant, the 95% highest-density interval of the distribution of their response latencies was computed, and latencies outside this interval were eliminated.

Analyses examined whether individual differences in anxiety or fear predicted differences in response latencies for correctly discriminated targets presented proximal to cues signalling negative pictures, as compared to non-negative pictures. Analyses also examined the internal reliability of the within-participant difference in mean response latencies between these targets (commonly labelled an “attentional bias index”), which indicates the reliability of the measurement of selective attentional responding.

Analysis of the relationship between individual differences in emotion and attention was conducted using linear mixed-effects regression models. For each model, the dependent variable was log-transformed response latencies^1^. Critical predictor variables represented the relative location of the probe to the negative cue (*probe location [negative cue location, non-negative* *cue location]*), variation in the questionnaire scores for the individual difference(s) of interest (*trait and state anxiety, social anxiety, or spider fear*), and their interaction term. Each model also included predictors to account for the effect of task progression (*trial number*), the effect of the preceding trial negative cue location (*previous* *negative cue location [same, different];* Talcott et al., 2022), the effect of whether the preceding trial was a picture trial or probe trial (*previous trial type [picture trial, probe trial]*), and the effect of the colour of the negative cue upon response latencies for probes in the location of negative cues as compared to non-negative cues (the interaction between *negative cue colour [blue, yellow] and probe location [negative cue location, non-negative* *cue location]*. Random effects included a random intercept effect of participant and a random slope effect of probe location (Barr, 2013). Effects from the regression model were evaluated through analysis of variance (ANOVA). Significant effects were inferred via *p*-values computed using Satterthwaite's method and appraised using model-predicted values. Estimated values for response latencies at specific levels of critical predictor variables, shown in figures, were computed after averaging over the levels of other factorial predictors in the model (marginal predictions). To evaluate potential speed–accuracy trade-off patterns, an identical logistic mixed-effects model was conducted on participant response accuracy. For brevity, reported results focus on the critical test(s) involving individual difference measures in the response latency models. The complete results of all analyses are available at the repository associated with this article (see Open Practices Statement).

The internal reliability of the measurement of biased selective attention for each task was assessed by computing the difference in mean response latency for probes in each cue location for each participant (labelled the “Attentional Bias to Negative Cues Index”), and then subjecting this index to a split-half reliability approach. To increase the robustness of these estimates, the index and its Spearman–Brown corrected correlation (*r*_SB_) was repeatedly computed across 5000 randomly selected split-halves (Parsons, 2021). The mean and 95% highest-density interval of the resulting distribution of split-half correlations served as the estimate and confidence interval of internal reliability.

Finally, many studies often amount to examining the relationship between individual differences and the Attentional Bias to Negative Cues Index. Therefore, analyses examined the association between measures of anxiety vulnerability and the Attentional Bias to Negative Cues Index in each study.

## Method and results unique to experiments

Features of the method that were unique to each experiment, and the results of analysis conducted for each experiment, are described below.

## Experiment 1: Trait and state anxiety

### Method

#### Participants

Two hundred and thirteen participants provided valid data. Sixty-five participants failed the negative cue comprehension check on one or more trials blocks. A further 14 participants demonstrated a probe discrimination accuracy rate below the inclusion criterion. Descriptive statistics of the final sample of 134 participants are presented in Table 1.

**Table 1:** Descriptive statistics of participant demographic characteristics and anxiety vulnerability measures, for each experiment; [mean (SD), range]

Measure	Experiment 1 (*N* = 134)	Experiment 2 (*N* = 122)	Experiment 3 (*N* = 131)
Gender: Woman:Man:Other	81:53:0	74:46:2	88:41:2
Age (years)	20.40 (3.63), 18–39	19.39 (2.70), 17–31	20.04 (3.39), 17–36
STAI-S score (state anxiety)	38.55 (11.32), 20–76		
STAI-T score (trait anxiety)	46.16 (11.71), 20–68		
SIAS score (social anxiety)		31.30 (14.32), 1–63	
FSQ score (fear of spiders)			64.94 (33.63), 18–125
FBQ score (fear of butterflies)			28.56 (16.03), 18–102
Response latency			
Probe in negative location	938.29 (179.14), 585.58–1464.2	867.43 (166.19), 593.79–1436.30	933.10 (196.46), 631.26–1522.68
Probe in non-negative location	916.97 (189.79), 560.96–1449.18	872.77 (157.92), 590.18–1382.80	923.17 (187.47), 571.14–1427.98
Attentional bias to negative cues index	−21.33 (65.05), −197.75 to 192.05	5.35 (43.68), −142.53 to 134.72	−9.93 (56.87), −376.09 to 105.53

#### State-Trait Anxiety Scale

The Spielberger State-Trait Anxiety Inventory (STAI; Spielberger et al., 1983) was used to assess participants’ levels of state anxiety and trait anxiety. The STAI contains two scales designed to measure an individual’s level of state anxiety “right now” (STAI-S) and their level of trait anxiety “in general” (STAI-T). Scores on each scale can range from 20 to 80, with higher scores representing higher levels of anxiety. The internal reliability amongst participants for the STAI-S was α_Chronbach_ = .94, CI_95%_ [.92, .95] and for the STAI-T was α_Chronbach_ = .94, CI_95%_ [.92, .95].

#### Picture stimuli

The attention assessment task presented 128 negatively valenced pictures to represent negative information, and 128 positively valenced pictures to represent non-negative information^2^. Pictures were drawn from emotional scenes contained within the International Affective Pictures System (IAPS; Bradley & Lang, 2017). The images taken from the IAPS system depicted injury, violence, weapons, disgust, and aggression. Examples of non-negative images include scenes featuring happy people, landscapes, and pleasant animals. IAPS pictures have been validated by ratings on a nine-point scale, with lower scores indicating more a negative valence of the picture content. In the present experiment, the mean valence rating of negative pictures was *M*(*SD*) = 2.20 (0.30) and non-negative pictures was *M*(*SD*) = 7.36 (0.42). A list of the images used is available in the materials repository associated with this article.

#### Attention assessment task

The attention assessment task included 768 trials, comprising 512 picture trials and 256 probe trials evenly delivered across four trial blocks.

### Results

The internal reliability estimate of the Attentional Bias to Negative Cues Index was *r*_SB_ = .81, CI_95%_[.74, .86]. This indicated that the measurement of selective attention in the task had a very high degree of internal consistency. Descriptive statistics of response latencies are presented in Table 1.

**Fig. 2:**
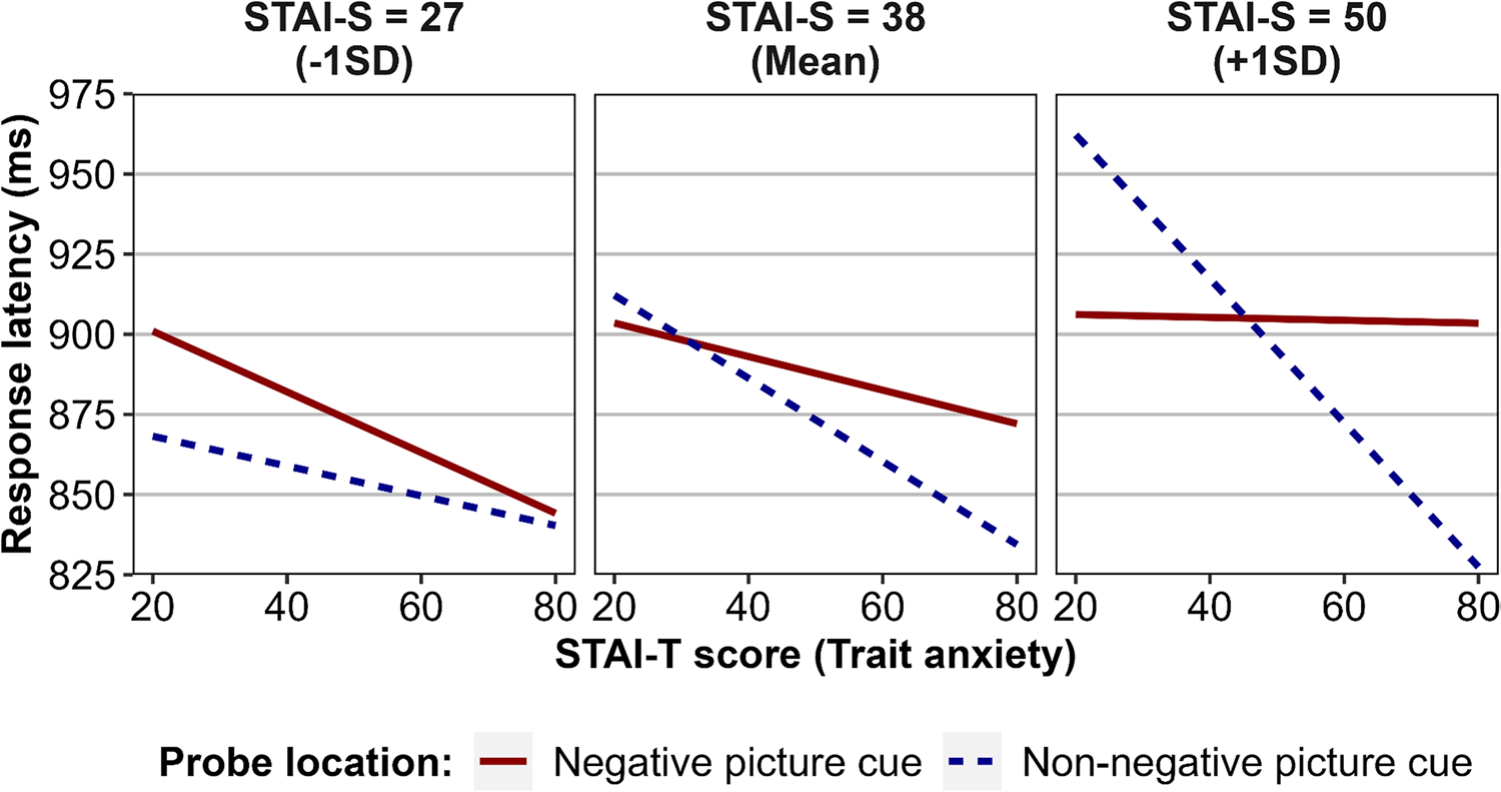
Experiment 1 regression model estimated values of response latency, in milliseconds (ms), for probes in threat cue and non-threat cue locations, across the possible range of STAI-T scores (trait anxiety) at discrete STAI-S scores (state anxiety). STAI-S scores are discretised at the sample mean and one standard deviation above and below the sample mean

The linear mixed-effects regression analysis revealed a significant main effect of probe location, *F*(1, 129.38) = 6.40, *p* = .013. Estimated marginal means (M_*EM*_) indicated that when averaging across all other effects, participants were slower to discriminate probes in the location of negative cues (*M*_*EM*_ = 889 ms) as compared to non-negative cues (*M*_*EM*_ = 877 ms). The regression analysis revealed a significant interaction effect involving probe location and STAI-S scores, *F*(1, 129.57) = 6.17, *p* = .014, which indicated that heightened state anxiety was associated with greater speed to discriminate probes in the location of negative cues. A significant effect involving probe location and STAI-T scores was also observed, *F*(1, 129.07) = 4.10, *p* = .045, which indicated that heightened trait anxiety was associated with greater speed to discriminate probes in the location of non-negative cues. Critically, these effects were subsumed by a significant three-way interaction effect involving probe-negative congruency, STAI-S scores, and STAI-T scores, *F*(1, 129.34) = 6.02, *p* = .015. The pattern arising from this effect is presented in Fig. 2. A Johnson-Neyman analysis revealed that the two-way interaction between STAI-T scores and probe location was statistically significant only when STAI-S scores were above 44. Analysis next fixed STAI-S scores at one standard deviation above the sample mean (STAI-S scores = 50) and found that, at this level of state anxiety, the effect of probe location became statistically significant (*p* < .05) when STAI-T scores were greater than 62. When state anxiety was fixed at one standard deviation below the mean (STAI-S scores = 27), the interaction between probe-congruency and STAI-T scores did not reach significance. The model evaluating response accuracy revealed no effects related to probe location or STAI-T or STAI-S scores.

Finally, a simple linear regression model was computed to examine the degree that individual differences in STAI-S scores and STAI-T scores, and their interaction, predicted Attentional Bias to Negative Cues Index scores. The regression analysis revealed a significant interaction effect, *b* = −.011, *t* = −2.36, *p* = .020, *r*^2^ = .043, Δ*r*^2^ = .041. Johnson-Neyman analysis revealed that the association between STAI-T scores and Attentional Bias to Negative Cues Index scores was negative and statistically significant when STAI-S scores were greater than 48.

Thus, these results indicated that the measurement of selective attention to negative cues exhibited a high degree of internal reliability, and that heightened levels of trait anxiety were associated with heightened selective attention away from cues signalling negative information when participants reported at moderate or higher levels of state anxiety.

## Experiment 2: Social anxiety

### Method

#### Participants

One hundred and ninety-two participants provided valid data. Sixty-four of these participants failed the negative cue comprehension check on one or more trials blocks. A further six participants demonstrated a probe discrimination accuracy rate below the inclusion criterion. Descriptive statistics of the final sample of 122 participants are presented in Table 1.

#### Social Interaction Anxiety Scale

The Social Interaction Anxiety Scale (SIAS; Mattick & Clarke, 1998) was used to assess participants’ social anxiety vulnerability. The 20-item scale requires participants to report on their emotional experiences in social situations. Scores on the SIAS can range from 0 to 80, with higher scores representing higher levels of social anxiety vulnerability. The internal reliability amongst participants was α_Chronbach_ = .93, CI_95%_ [.91, .95].

#### Picture stimuli

The attention assessment task presented pictures depicting 36 male faces with angry expressions and 36 male faces with neutral expressions drawn from the Karolinska Directed Emotional Faces picture set (KDEF; Lundqvist et al., 1998). A list of the images used is available in the materials repository associated with this article.

#### Attention assessment task

The attention assessment task included 864 trials, comprising 576 picture trials and 288 probe trials evenly delivered across four trial blocks.

### Results

**Fig. 3:**
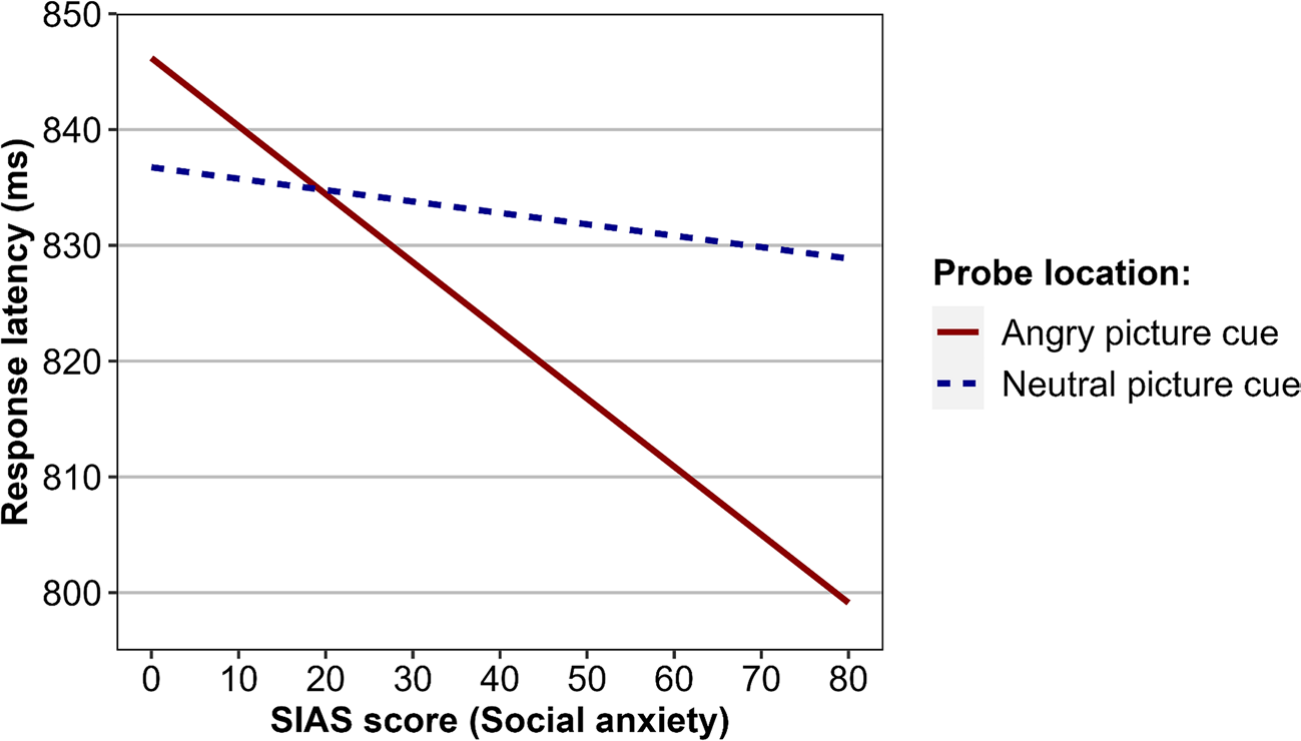
Experiment 2 regression model estimated values of response latency, in milliseconds (ms), for probes in threat cue and non-threat cue locations, across the possible range of SIAS scores (social anxiety)

The internal reliability estimate of the Attentional Bias to Negative Cues Index was *r*_SB_ = .69, CI_95%_[.57, .78]. This indicated that the measurement of selective attention in the task had a good degree of internal consistency. Descriptive statistics of response latencies are presented in Table 1. The linear mixed-effects regression analysis did not reveal a significant main effect of probe location, *F*(1, 118.78) = 1.40, *p* = .24, indicating that, when averaging across all other effects, participants did not differ significantly in their response latencies for probes in the location of negative cues (*M*_*EM*_ = 828 ms) as compared to non-negative cues (*M*_*EM*_ = 834 ms). The regression analysis did reveal a significant interaction effect involving probe location and SIAS scores, *F*(1, 118.86) = 4.42, *p* = .038, which indicated that social anxiety was associated with variation in the speed to discriminate probes in the location of negative cues relative to non-negative cues. The pattern of predicted values related to this effect are presented in Fig. 3. Simple slopes analysis reveals the effect of probe location fixed at one standard deviation above the sample mean (SIAS score = 45), *t* = 2.46 *p* = .015, but not when fixed one standard deviation below the mean (SIAS score = 17), *t* = 0.28, *p* = .78. A Johnson-Neyman analysis revealed that the effect of probe location was statistically significant (*p* < .05) when SIAS scores were greater than 34. The model evaluating response accuracy revealed no effects related to probe location or SIAS scores.

Finally, a simple linear regression model was computed to examine the degree that individual differences in SIAS scores predicted Attentional Bias to Negative Cues Index scores. The regression analysis revealed a non-significant effect, *b* = 0.52, *t* = 1.90, *p* = .060, *r*^2^ = .029. This indicated that level of social anxiety was not associated with a difference-score that indexed biased attention to cues signalling angry faces.

Thus, these results indicated that the measurement of selective attention to negative cues exhibited a good degree of internal reliability, and that heightened levels of social anxiety were associated with greater selective attention towards cues signalling negative information.

## Experiment 3: Spider fear

### Method

#### Participants

One hundred and eighty-four participants provided valid data. Forty-seven participants failed the negative cue comprehension check on one or more trials blocks. A further six participants demonstrated accuracy below the inclusion criterion. Descriptive statistics of the final sample of 131 participants are presented in Table 1.

#### Fear of spiders and butterflies questionnaires

The Fear of Spiders Questionnaire (Szymanski & O’Donohue, 1995) was used to measure spider fear. The 18-item scale requires participants to rate the degree to which they experience behavioural, physiological, and cognitive symptoms of spider fear. Scores on the FSQ can range from 18 to 126, with higher scores reflecting greater spider fear. The Fear of Spiders Questionnaire has been demonstrated to hold construct validity amongst undergraduate student populations (Muris & Merckelbach, 1996; Szymanski & O’Donohue, 1995). The internal reliability amongst participants was α_*Cronbach*_ = .98, CI_95%_[.97, .98].

As pictures of butterflies were used to represent a non-feared stimulus, it was considered appropriate to statistically control for the effect of fear of butterflies when analysing biased attention to spider cues stimuli. As such, a Fear of Butterflies Questionnaire was developed that emulated the Fear of Spiders Questionnaire but replaced reference to spiders with reference to butterflies. The internal reliability amongst participants was α_*Cronbach*_ = .95, CI_95%_[.92, .97].

#### Picture stimuli

The attention assessment task presented 96 colour pictures of spiders and 96 colour pictures of butterflies. Each picture displayed the animal atop a white background with the width of the animal sized to extend to the width of the picture. These spider and butterfly pictures have been demonstrated to be differentially valenced amongst spider-fearful individuals (Basanovic et al., 2017, 2019, 2022). The images used are available in the materials repository associated with this article.

#### Attention assessment task

The attention assessment task included 864 trials, comprising 576 picture presentation trials and 288 probe presentation trials evenly delivered across six trial blocks.

### Results

**Fig. 4:**
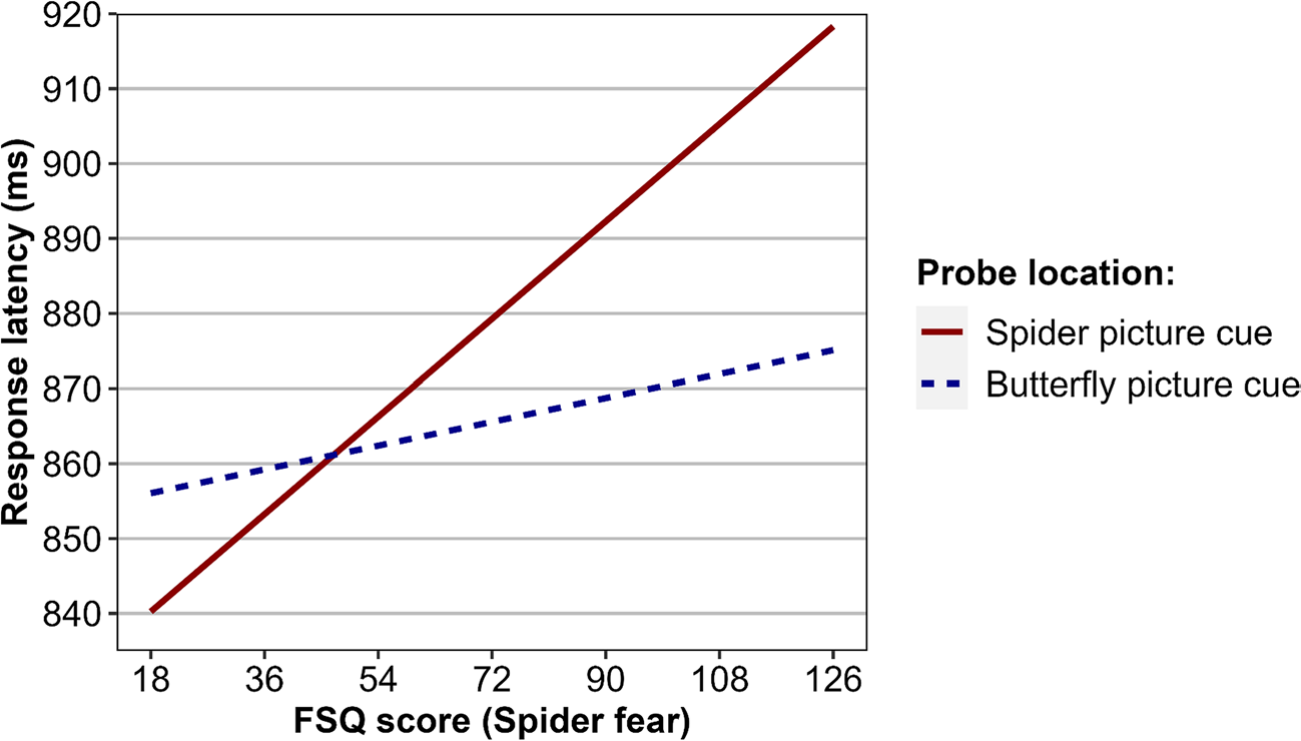
Experiment 3 regression model estimated values of response latency, in milliseconds (ms), for probes in threat cue and non-threat cue locations, across the possible range of FSQ scores (spider fear)

The internal reliability estimate of the Attentional Bias to Negative Cues Index was *r*_SB_ = .78, CI_95%_[.70, .84]. This indicated that the measurement of selective attention in the task had a high degree of internal consistency. Descriptive statistics of response latencies are presented in Table 1. The linear mixed-effects regression model in Experiment 3 included an interaction effect between the within-participants factor *probe-location* and Fear of Butterflies Questionnaire scores to account for the effect that fear of butterflies may have upon response latencies for probes in the location of butterfly cues as compared to spider cues. The analysis did not reveal a significant main effect of probe location, *F*(1, 130.31) = 3.74, *p* = .055, indicating that, when averaging across all other effects, participants did not differ significantly in their response latencies for probes in the location of negative cues (*M*_*EM*_ = 875 ms) as compared to non-negative cues (*M*_EM_ = 865 ms). The regression analysis did reveal a significant interaction effect involving probe location and FSQ scores, *F*(1, 127.28) = 13.69, *p* < .001, which indicated that spider fear was associated with variation in the speed to discriminate probes in the location of negative cues relative to non-negative cues. The pattern of predicted values related to this effect are presented in Fig. 4. Simple slopes analysis revealed that the regression slope of probe location was significant when FSQ scores were fixed at one standard deviation above the sample mean (FSQ score = 98), *t* = 2.51, *p* = .013, and significant, but in the reverse direction, when fixed at one standard deviation below the mean (FSQ score = 31), *t* = −2.06, *p* = .042. A Johnson–Neyman analysis revealed that the effect of probe location was statistically significant (*p* < .05) when FSQ scores were less than 33 or greater than 87. The model evaluating response accuracy revealed no effects related to probe location, FSQ scores, or FBQ scores.

Finally, a simple linear regression model was computed to examine the degree to which individual differences in FSQ scores predicted Attentional Bias to Negative Cues Index scores independently of FBQ scores. The regression analysis revealed a significant effect, *b* = −0.54, *t* = −3.36, *p* = .001, *r*^2^ = .096. This indicated that level of spider fear was associated with a difference-score that indexed biased attention to cues signalling spider pictures.

Thus, these results indicated that the measurement of selective attention to negative cues exhibited a high degree of internal reliability, and that heightened spider fear was associated with greater selective attention away from cues signalling negative information.

## Discussion

The aim of the present study was to determine whether biases in attentional responding to cues signalling the imminent location of negative information are related to anxiety vulnerability, and whether such biases can be measured with acceptable reliability. To do so, the study used anxiety vulnerability as an exemplar emotional vulnerability and examined whether anxiety vulnerability was associated with attentional responding to signals of negative information, and whether an index of the attentional bias for cues signalling negative information was assessed with satisfactory measurement reliability.

Results revealed wide-ranging individual variation in patterns of attentional preference for cues signalling emotional information. This suggests that attentional responding to cues signalling emotional information varies across individuals. Results also revealed that the method could measure this process with an acceptable to high level of internal reliability. This indicates that the method holds acceptable psychometric qualities for research that would explore the relationship between emotional vulnerability and attention to signals of emotional information.

The study also found links between attentional responses to cues signalling negative information and individual differences in state and trait anxiety, social anxiety, and spider fear. These associations varied across different anxiety domains. In Experiment 1, under moderate or high levels of state anxiety, individuals with greater trait anxiety showed more attentional avoidance of cues indicating negative scenes compared to non-negative scenes. Experiment 2 showed that individuals with higher levels of social anxiety allocated greater attention towards cues signalling the location of angry faces, as compared to neutral faces. In Experiment 3, individuals with a greater fear of spiders showed greater attentional avoidance of cues signalling the location of spiders, as compared to butterflies.

The present findings indicate that the present task can yield an appropriately reliable measure of biased attention to cues signalling negative information. A clear question then is why the measurement of attentional biases to stimuli that signal negative information appear to be more reliable than attentional biases to negative information itself. While yet untested, there are plausible explanations. For example, when assessing attention to emotional stimuli, both emotional effects but also extraneous stimulus attributes, such as complexity, colour, ease of valence recognition, or arousal, and extraneous between-participant effects such as speed at which emotional content is processed, each influence the degree to which attentional biases are expressed. This adds to measurement error and so undermines the reliability of the effect of interest. In contrast, the task used herein eliminates these sources of measurement error.

Another relevant query is whether biased attentional responding to signals of emotional information and to emotional information itself are driven by the same mechanisms. Interestingly, Experiment 1 found an anxiety-linked avoidance of negative cues, which contrasts with the commonly reported pattern of anxiety-linked attention towards negative information (Bar-Haim et al., 2007). Experiment 2 produced results consistent with typical patterns of social anxiety-linked attentional responses to socially negative information (Bantin et al., 2016). Experiment 3, however, contradicted research showing attentional vigilance for spider stimuli (Lipp & Derakshan, 2005), but agreed with studies demonstrating fear-linked attentional avoidance of spider stimuli (Hermans et al., 1999; Pflugshaupt et al., 2005, 2007). However, while in the present study patterns of attention sometimes diverged from research investigating attention to emotional information itself, some evidence suggests that these processes might be related. Gladwin et al. (2019) had participants complete a task designed to manipulate attention towards, or away from, cues signalling negative information and subsequently observed a difference in how the groups responded to negative information that aligned with the manipulation. Such findings suggest that common mechanisms might govern attentional responses to emotional information and information that signals them. Interestingly, if these processes are related, then attentional responses to cued stimuli could offer a more reliable method for investigating the relationship between attentional processing of emotional information and emotional vulnerabilities.

If research reveals attention to signals of emotional information to be unrelated to attention to emotional information itself, then this would open the door to research that independently considers these processes when testing theories of negative emotion vulnerability. For example, theorists have proposed that individuals with depression are characterised by a cognitive system that is motivated to avoid positive information (Winer & Salem, 2016). While data from attentional-probe tasks have revealed evidence consistent with this proposal as shown by reduced attentional preference for positive information, such findings could arise from a motivation to avoid positive information or from reduced salience or recognition of positive information. By evaluating attention to cues signalling imminent positive information, researchers could discriminate these possibilities. If depressed individuals show avoidance of signals of positive information, this will provide evidence consistent with the explanation that the cognitive system is motivated to avoid positive information, rather than impaired in processing it.

Another avenue for research could be to determine the impact of manipulating attention to signals of emotional information upon emotional vulnerability. Many studies have investigated whether the manipulation of attentional responding to emotional information itself impacts state emotion and emotional vulnerability, and positive findings in this field have prompted researchers to try to translate these methods for therapeutic aims (Basanovic et al., 2020; Browning et al., 2012; Clarke et al., 2014; Grafton et al., 2017; Hallion & Ruscio, 2011; MacLeod & Clarke, 2015; Todd et al., 2016; Wiers et al., 2015). If manipulation of attention to cues signalling emotional information is found to influence emotional vulnerability, this would reveal a novel approach to reducing negative emotional vulnerability.

When considering the present findings, it is important to note that the procedures used in each experiment assess attentional deployment amongst stimulus cues only at 500 ms following their onset. Therefore, the current task parameters cannot show how attentional responses to emotional cues change at earlier or later times. Some studies suggest that patterns of attentional responding to cues signalling emotional information do not vary within individuals between 200 ms and 1000 ms after stimulus onset (Gladwin et al., 2019). However, it is unknown whether the association between attentional processes and emotional vulnerability is consistent over time.

For the moment, however, the present study has determined that biases in attentional responding to cues signalling negative information are related to anxiety vulnerability across three anxiety domains, and that such biases can be measured with acceptable reliability. It is hoped that these findings will provide new opportunities for understanding the relationship between emotional vulnerability and attention.

## Data Availability

The task materials, data, and analyses reported in this article are available at https://osf.io/k82tg/.

## Footnotes

1 Log-transformed response latencies were used, as they resulted in closer alignment of the model predictions and the observed data. Outputs of models using non-transformed response latencies resulted in the same pattern of results.

2 Dominant approaches to evaluating and theorising anxiety-linked differences in attentional biases to negative information have typically not drawn a distinction between negatively valenced information and information depicting social or physical threats. However, it is notable that negative information is not necessarily threatening, and recent research has suggested that images showing negatively valenced non-threat information are not necessarily processed identically to images showing threatening information (March et al., 2020).

## References

[CR1] Bantin T, Stevens S, Gerlach AL, Hermann C (2016). What does the facial dot-probe task tell us about attentional processes in social anxiety? A systematic review. Journal of Behavior Therapy and Experimental Psychiatry.

[CR2] Bar-Haim Y, Lamy D, Pergamin L, Bakermans-Kranenburg MJ, van IJzendoorn MH (2007). Threat-related attentional bias in anxious and nonanxious individuals: A meta-analytic study. Psychological Bulletin.

[CR3] Barr, D. J. (2013). Random effects structure for testing interactions in linear mixed-effects models. *Frontiers in Psychology*, *0*, 328. 10.3389/FPSYG.2013.00328 10.3389/fpsyg.2013.00328 PMC3672519 23761778

[CR4] Basanovic J, MacLeod C (2017). Does anxiety-linked attentional bias to threatening information reflect bias in the setting of attentional goals, or bias in the execution of attentional goals?. Cognition and Emotion.

[CR5] Basanovic J, Dean L, Riskind JH, MacLeod C (2017). Direction of stimulus movement alters fear-linked individual differences in attentional vigilance to spider stimuli. Behaviour Research and Therapy.

[CR6] Basanovic J, Dean L, Riskind JH, MacLeod C (2019). High Spider-Fearful and Low Spider-Fearful Individuals Differentially Perceive the Speed of Approaching, but not Receding. Spider Stimuli. Cognitive Therapy and Research.

[CR7] Basanovic J, Grafton B, Ford A, Hirani V, Glance D, MacLeod C, Almeida OP (2020). Cognitive bias modification to prevent depression (COPE): Results of a randomised controlled trial. Psychological Medicine.

[CR8] Basanovic J, Kaiko I, MacLeod C (2021). Change in Attentional Control Predicts Change in Attentional Bias to Negative Information in Response to Elevated State Anxiety. Cognitive Therapy and Research.

[CR9] Basanovic J, Page J, MacLeod C (2022). The attenuation of spider avoidance action tendencies in spider-fearful individuals and its impact on explicit evaluation of spider stimuli. Behaviour Research and Therapy.

[CR10] Beck AT, Clark DA (1997). An information processing model of anxiety: Automatic and strategic processes. Behaviour Research and Therapy.

[CR11] Bradley BP, Mogg K, White J, Groom C, de Bono J (1999). Attentional bias for emotional faces in generalized anxiety disorder. The British Journal of Clinical Psychology / the British Psychological Society.

[CR12] Browning M, Holmes EA, Charles M, Cowen PJ, Harmer CJ (2012). Using attentional bias modification as a cognitive vaccine against depression. Biological Psychiatry.

[CR13] Chapman, A., Devue, C., & Grimshaw, G. M. (2017). Fleeting reliability in the dot-probe task. *Psychological Research*, *0*(0), 1–13. 10.1007/s00426-017-0947-6 10.1007/s00426-017-0947-6 29159699

[CR14] Cisler JM, Koster EHW (2011). Mechanisms of attentional biases towards threat in anxiety disorder: An integrative review. Clinical Psychology Review.

[CR15] Clarke PJF, Notebaert L, MacLeod C (2014). Absence of evidence or evidence of absence: Reflecting on therapeutic implementations of attentional bias modification. BMC Psychiatry.

[CR16] Clarke PJF, Marinovic W, Todd J, Basanovic J, Chen NTM, Notebaert L (2020). What is attention bias variability? Examining the potential roles of attention control and response time variability in its relationship with anxiety. Behaviour Research and Therapy.

[CR17] Cooper SR, Gonthier C, Barch DM, Braver TS (2017). The role of psychometrics in individual differences research in cognition: A case study of the AX-CPT. Frontiers in Psychology.

[CR18] Dondzilo L, Basanovic J (2023). Body dissatisfaction and selective attention to thin-ideal bodies: The moderating role of attentional control. Body Image.

[CR19] Dondzilo, L., Basanovic, J., Grafton, B., Bell, J., Turnbull, G., & MacLeod, C. (2021). A serial mediation model of attentional engagement with thin bodies on body dissatisfaction: The role of appearance comparisons and rumination. *Current Psychology*, *2017*. 10.1007/s12144-021-01574-1

[CR20] Georgiades J, Cusworth K, MacLeod C, Notebaert L (2021). The relationship between worry and attentional bias to threat cues signalling controllable and uncontrollable dangers. PLOS ONE.

[CR21] Gladwin TE (2019). Spatial anticipatory attentional bias for alcohol: A preliminary report on reliability and associations with risky drinking. Alcoholism and Drug Addiction.

[CR22] Gladwin TE, Vink M (2020). Spatial anticipatory attentional bias for threat: Reliable individual differences with RT-based online measurement. Consciousness and Cognition.

[CR23] Gladwin TE, Möbius M, McLoughlin S, Tyndall I (2019). Anticipatory versus reactive spatial attentional bias to threat. British Journal of Psychology.

[CR24] Gladwin TE, Banic M, Figner B, Vink M (2020). Predictive cues and spatial attentional bias for alcohol: Manipulations of cue-outcome mapping. Addictive Behaviors.

[CR25] Gladwin, T. E., Halls, M., & Vink, M. (2021). Experimental control of conflict in a predictive visual probe task: Highly reliable bias scores related to anxiety. *Acta Psychologica*, *218*. 10.1016/J.ACTPSY.2021.103357 10.1016/j.actpsy.2021.103357 34175671

[CR26] Grafton B, MacLeod C, Rudaizky D, Holmes EA, Salemink E, Fox E, Notebaert L (2017). Confusing procedures with process when appraising the impact of cognitive bias modification on emotional vulnerability. British Journal of Psychiatry.

[CR27] Hallion LS, Ruscio AM (2011). A meta-analysis of the effect of cognitive bias modification on anxiety and depression. Psychological Bulletin.

[CR28] Hermans D, Vansteenwegen D, Eelen P (1999). Eye Movement Registration as a Continuous Index of Attention Deployment: Data from a Group of Spider Anxious Students. Cognition & Emotion.

[CR29] Joormann J, Gotlib IH (2007). Selective attention to emotional faces following recovery from depression. Journal of Abnormal Psychology.

[CR30] Koster EHW, Crombez G, Verschuere B, Vanvolsem P, De Houwer J (2007). A time-course analysis of attentional cueing by threatening scenes. Experimental Psychology.

[CR31] Koster E, Crombez G, Van Damme S, Verschuere B, De Houwer J (2005). Signals for threat modulate attentional capture and holding: Fear-conditioning and extinction during the exogenous cueing task. Cognition and Emotion.

[CR32] Kruijt A-W, Parsons S, Fox E (2019). A meta-analysis of bias at baseline in RCTs of attention bias modification: No evidence for dot-probe bias towards threat in clinical anxiety and PTSD. Journal of Abnormal Psychology.

[CR33] Bradley, M. M., & Lang, P. J. (2017). International Affective Picture System. In V. Zeigler-Hill & T. K. Shackelford (Eds.), Encyclopedia of Personality and Individual Differences (pp. 1–4). Springer International Publishing. 10.1007/978-3-319-28099-8_42-1

[CR34] LeMoult J, Gotlib IH (2019). Depression: A cognitive perspective. Clinical Psychology Review.

[CR35] Lipp OV, Derakshan N (2005). Attentional bias to pictures of fear-relevant animals in a dot probe task. Emotion.

[CR36] Liu J, Shen K, Li H (2019). How state anxiety and attentional bias interact with each other: The moderating effect of cognitive appraisal. Attention, Perception, and Psychophysics.

[CR37] Lundqvist, D., Flykt, A., & Öhman, A. (1998). The Karolinska Directed Emotional Faces - KDEF, CD ROM from Department of Clinical Neuroscience, Psychology section, Karolinska Institutet, ISBN 91-630-7164-9. 10.1037/t27732-000

[CR38] MacLeod C, Clarke PJF (2015). The Attentional Bias Modification Approach to Anxiety Intervention. Clinical Psychological Science.

[CR39] MacLeod C, Mathews A (1988). Anxiety and the Allocation of Attention to Threat. The Quarterly Journal of Experimental Psychology Section A.

[CR40] MacLeod C, Mathews A, Tata P (1986). Attentional bias in emotional disorders. Journal of Abnormal Psychology.

[CR41] MacLeod C, Rutherford E, Campbell L, Ebsworthy G, Holker L (2002). Selective attention and emotional vulnerability: Assessing the causal basis of their association through the experimental manipulation of attentional bias. Journal of Abnormal Psychology.

[CR42] March, D. S., Olson, M. A., & Gaertner, L. (2020). Lions, and tigers, and implicit measures, oh my! implicit assessment and the valence vs. Threat Distinction*. Social Cognition,**38*(Supplement), s154–s164. 10.1521/soco.2020.38.supp.s154

[CR43] Mathews A, Mackintosh B (1998). A Cognitive Model of Selective Processing in Anxiety. Therapy.

[CR44] Mattick R, Clarke C (1998). Development and Validation of Measure of Social Phobia Scrutiny Fear and Social Interaction Anxiety. Behavior Research and Therapy.

[CR45] Mazidi M, Grafton B, Basanovic J, MacLeod C (2021). Attentional control moderates the relationship between social anxiety and selective attentional responding to negative social information: Evidence from objective measures of attentional processes. Cognition and Emotion.

[CR46] Merckelbach H, Kenemans JL, Dijkstra A, Schouten E (1993). No attentional bias for pictoral stimuli in spider-fearful subjects. Journal of Psychopathology and Behavioral Assessment.

[CR47] Mogg K, Bradley BP (1998). A cognitive-motivational analysis of anxiety. Behaviour Research and Therapy.

[CR48] Mogg K, Bradley BP (2006). Time course of attentional bias for fear-relevant pictures in spider-fearful individuals. Behaviour Research and Therapy.

[CR49] Mogg K, Bradley BP, Hallowell N (1994). Attentional bias to threat: Roles of trait anxiety, stressful events, and awareness. The Quarterly Journal of Experimental Psychology A, Human Experimental Psychology.

[CR50] Mogg K, Bradley B, Miles F, Dixon R (2004). Time course of attentional bias for threat scenes: Testing the vigilance-avoidance hypothesis. Cognition & Emotion.

[CR51] Muris P, Merckelbach H (1996). A comparison of two spider fear questionnaires. Journal of Behavior Therapy and Experimental Psychiatry.

[CR52] Notebaert L, Tilbrook M, Clarke PJF, MacLeod C (2017). When a Bad Bias Can Be Good: Anxiety-Linked Attentional Bias to Threat in Contexts Where Dangers Can Be Avoided. Clinical Psychological Science.

[CR53] Notebaert L, Georgiades JV, Herbert M, Grafton B, Parsons S, Fox E, MacLeod C (2020). Trait anxiety and the alignment of attentional bias with controllability of danger. Psychological Research.

[CR54] Parsons S (2021). splithalf: Robust estimates of split half reliability. Journal of Open Source Software.

[CR55] Parsons S, Kruijt A-W, Fox E (2019). Psychological Science Needs a Standard Practice of Reporting the Reliability of Cognitive-Behavioral Measurements. Advances in Methods and Practices in Psychological Science.

[CR56] Pflugshaupt T, Mosimann UP, Von Wartburg R, Schmitt W, Nyffeler T, Müri RM (2005). Hypervigilance-avoidance pattern in spider phobia. Journal of Anxiety Disorders.

[CR57] Pflugshaupt T, Mosimann UP, Schmitt WJ, von Wartburg R, Wurtz P, Lüthi M, Nyffeler T, Hess CW, Müri RM (2007). To look or not to look at threat?. Scanpath differences within a group of spider phobics. Journal of Anxiety Disorders.

[CR58] Pishyar R, Harris LM, Menzies RG (2004). Attentional bias for words and faces in social anxiety. Anxiety, Stress & Coping.

[CR59] Ponterotto JG, Ruckdeschel DE (2007). An Overview of Coefficient Alpha and a Reliability Matrix for Estimating Adequacy of Internal Consistency Coefficients with Psychological Research Measures. Perceptual and Motor Skills.

[CR60] Rinck M, Becker ES (2006). Spider fearful individuals attend to threat, then quickly avoid it: Evidence from eye movements. Journal of Abnormal Psychology.

[CR61] Rudaizky D, Basanovic J, MacLeod C (2014). Biased attentional engagement with, and disengagement from, negative information: Independent cognitive pathways to anxiety vulnerability?. Cognition and Emotion.

[CR62] Schmidt FL, Hunter JE (1999). Theory Testing and Measurement Error. Intelligence.

[CR63] Schmukle SC (2005). Unreliability of the dot probe task. European Journal of Personality.

[CR64] Shaw M, Cloos LJR, Luong R, Elbaz S, Flake JK (2020). Measurement practices in large-scale replications: Insights from Many Labs 2. Canadian Psychology / Psychologie Canadienne.

[CR65] Spielberger CD, Gorsuch RL, Lushene R, Vagg PR, Jacobs GA (1983). Manual for the State-Trait Anxiety Inventory.

[CR66] Szymanski J, O’Donohue W (1995). Fear of Spiders Questionnaire. Journal of Behavior Therapy and Experimental Psychiatry.

[CR67] Talcott TN, Levy AP, Gaspelin N (2022). Covert attention is attracted to prior target locations: Evidence from the probe paradigm. Attention, Perception, & Psychophysics.

[CR68] Todd J, Sharpe L, Johnson A, Nicholson Perry K, Colagiuri B, Dear BF (2015). Towards a new model of attentional biases in the development, maintenance, and management of pain. Pain.

[CR69] Todd J, Sharpe L, Colagiuri B (2016). Attentional bias modification and pain: The role of sensory and affective stimuli. Behaviour Research and Therapy.

[CR70] Todd J, van Ryckeghem DML, Sharpe L, Crombez G (2018). Attentional bias to pain-related information: A meta-analysis of dot-probe studies. Health Psychology Review.

[CR71] Van Bockstaele, B., Lamens, L., Salemink, E., Wiers, R. W., Bögels, S. M., & Nikolaou, K. (2019). Reliability and validity of measures of attentional bias towards threat in unselected student samples: Seek, but will you find? *Cognition and Emotion*, *0*(0), 1–12. 10.1080/02699931.2019.1609423 10.1080/02699931.2019.1609423 31044648

[CR72] Waechter S, Stolz JA (2015). Trait Anxiety, State Anxiety, and Attentional Bias to Threat: Assessing the Psychometric Properties of Response Time Measures. Cognitive Therapy and Research.

[CR73] Wiers CE, Stelzel C, Gladwin TE, Park SQ, Pawelczack S, Gawron CK, Stuke H, Heinz A, Wiers RW, Rinck M, Lindenmeyer J, Walter H, Bermpohl F (2015). Effects of Cognitive Bias Modification Training on Neural Alcohol Cue Reactivity in Alcohol Dependence. American Journal of Psychiatry.

[CR74] Williams JMG, Watts FN, MacLeod C, Mathews A (1988). Cognitive psychology and emotional disorders.

[CR75] Winer ES, Salem T (2016). Reward devaluation: Dot-probe meta-analytic evidence of avoidance of positive information in depressed persons. Psychological Bulletin.

[CR76] Yiend J, Mathews A (2001). Anxiety and attention to threatening pictures. The Quarterly Journal of Experimental Psychology Section A.

